# Lactate Clearance Predicts Good Neurological Outcomes in Cardiac Arrest Patients Treated with Extracorporeal Cardiopulmonary Resuscitation

**DOI:** 10.3390/jcm8030374

**Published:** 2019-03-18

**Authors:** Christian Jung, Sandra Bueter, Bernhard Wernly, Maryna Masyuk, Diyar Saeed, Alexander Albert, Georg Fuernau, Malte Kelm, Ralf Westenfeld

**Affiliations:** 1 Division of Cardiology, Pulmonology and Vascular Medicine, Medical Faculty, University Hospital Düsseldorf, Heinrich-Heine-University Düsseldorf, D-40225 Düsseldorf, Germany; sandra.bueter@med.uni-duesseldorf.de (S.B.); maryna.masyuk@med.uni-duesseldorf.de (M.M.); malte.kelm@med.uni-duesseldorf.de (M.K.); ralf.westenfeld@med.uni-duesseldorf.de (R.W.); 2 Department of Internal Medicine, Department of Cardiology, Medical Faculty, Friedrich-Schiller-University Jena, 07743 Jena, Germany; 3 Clinic of Internal Medicine II, Department of Cardiology, Paracelsus Medical University of Salzburg, 5020 Salzburg, Austria; b.wernly@salk.at; 4 Division of Cardiovascular Surgery, Medical Faculty, University Hospital Düsseldorf, Heinrich-Heine-University Düsseldorf, D-40225 Düsseldorf, Germany; diyar.saeed@med.uni-duesseldorf.de (D.S.); alexander.albert@med.uni-duesseldorf.de (A.A.); 5 Clinic for Internal Medicine/Cardiology/Angiology/Intensive Care Medicine, University Heart Center Luebeck, 23562 Lübeck, Germany; georg.fuernau@uksh.de

**Keywords:** cardiac arrest, acute myocardial infarction, neurological outcome, lactate clearance

## Abstract

Background: We evaluated critically ill patients undergoing extracorporeal cardiopulmonary resuscitation (ECPR) due to cardiac arrest (CA) with respect to baseline characteristics and laboratory assessments, including lactate and lactate clearance for prognostic relevance. Methods: The primary endpoint was 30-day mortality. The impact on 30-day mortality was assessed by uni- and multivariable Cox regression analyses. Neurological outcome assessed by Glasgow Outcome Scale (GOS) was pooled into two groups: scores of 1–3 (bad GOS score) and scores of 4–5 (good GOS score). Results: A total of 93 patients were included in the study. Serum lactate concentration (hazard ratio (HR) 1.09; 95% confidence interval (CI) 1.04–1.13; *p* < 0.001), hemoglobin, (Hb; HR 0.87; 95% CI 0.79–0.96; *p* = 0.004), and catecholamine use were associated with 30-day-mortality. In a multivariable model, only lactate clearance (after 6 h; OR 0.97; 95% CI 0.94–0.997; *p* = 0.03) was associated with a good GOS score. The optimal cut-off of lactate clearance at 6 h for the prediction of a bad GOS score was at ≤13%. Patients with a lactate clearance at 6 h ≤13% evidenced higher rates of bad GOS scores (97% vs. 73%; *p* = 0.01). Conclusions: Whereas lactate clearance does not predict mortality, it was the sole predictor of good neurological outcomes and might therefore guide clinicians when to stop ECPR.

## 1. Introduction

Cardiac arrest (CA) is a major cause of sudden death in developed countries, leading to poor patient outcomes [1]. Although organizational advancements have been made in cardiopulmonary resuscitation (CPR), especially with early defibrillation and minimization of the delay in starting CPR, mortality due to out-of-hospital cardiac arrest (OHCA) and in-hospital cardiac arrest (IHCA) remain high [2]. Survival rates have only slightly improved over the past few decades [3].

Extracorporeal membrane oxygenation in the scenario of CPR establishing extracorporeal CPR (ECPR) has been proven to be a therapeutic option in refractory CA [4]. ECPR restores oxygen delivery by providing sufficient perfusion of vital organs and allowing the treatment of the cause of CA [5]. Thus, current American Heart Association guidelines recommend that ECPR should be considered in CA; however, large-scale studies are lacking [6]. 

A systematic review analyzed a total of 833 patients in 20 studies treated with ECPR. Although there were differences in the timepoints of the reported outcomes, the overall acute survival rate was 22% and 21% after three months [7]. Ha and colleagues identified witnessed arrest, bystander CPR, and successful revascularization as independent predictors of survival in patients with OHCA [8]. 

Recently, Dennis et al. found that in a mixed cohort of patients treated with ECPR for OHCA and IHCA, serum lactate levels before ECPR implementation predicted clinical outcomes [9]. This is of special interest, because quick decision making either to implement ECPR or to stop futile therapy escalation is crucial for adequate resource allocation. Serum lactate levels have been proven to predict outcomes in different states of shock associated with organ failure, such as cardiogenic and septic shock indicating inadequate tissue perfusion [10]. Recently, lactate clearance has emerged as an important variable [11]. Lactate clearance refers to serial lactate measurements in the early phase of treatment and hence describes excess lactate formation in relation to clearance and the extent of early metabolic recovery following the restoration of circulation. While early, high lactate clearance is associated with improved outcome in sepsis and following CA, low lactate clearance is associated with increased mortality [12,13].

Anemia is known to be associated with poor outcomes in ischemic heart disease, cerebrovascular disease, and patients undergoing surgery [14]. Thus, anemia might represent a potential parameter for risk stratification after CA [15].

Here, we investigated clinical outcomes and predictors of survival in patients following CA who underwent ECPR in two tertiary centers, and we evaluated baseline laboratory and easily available clinical parameters to assess lactate and its changes after 6, 12, and 24 h as prognostic parameters.

## 2. Methods

### 2.1. Study Patients

A total of 93 patients treated with ECPR due to IHCA and OHCA were included in this retrospective cohort study at the University hospitals in Jena and Duesseldorf, Germany, conducted from 2002 to 2013. The study was approved by the local institutional ethics boards (Jena: #2081, Duesseldorf: #5194). According to German regulations, no consent of the patients was needed in this retrospective study. 

### 2.2. Inclusion Criteria

Indication of and eligibility for ECPR following CA was evaluated by the staff cardiologist on duty in cooperation with the department of cardiovascular surgery. The majority of patients considered for ECPR were aged between 18 and 74 years, and only a few older patients were found to be eligible based on individual decisions. Failure to achieve ROSC (Return of spontaneous circulation) within twenty minutes of CPR qualified for ECPR according to guidelines. Patients who underwent cardiac surgery or resuscitation following interventions due to structural heart disease prior to ECPR implantation were not included in the analysis. Similarly, patients who did not survive longer than 6 h after ECPR implantation or whose ECPR running time was shorter than 6 h were excluded, because no lactate clearance was available. Irreversible preexisting neurological damages, malignant comorbidities, and an initial pH below 7.0 were defined as contraindications for ECPR implantation.

### 2.3. Data Collection

All the data were collected from patient charts and medical records through primary discharge, including laboratory parameters, complications, and therapy strategies. The neurological outcome was determined on day 30 according to the Glasgow Outcome Scale (GOS) encoded on a five-point scale from 1 (death) to 5 (good recovery). The given timepoints of the laboratory value measurements were within a 60 min windows (e.g., 6-hour lactate between 5:30 and 6:30).

### 2.4. Extracorporal Life Support (ECLS) System

Three different ECLS systems have been implemented: Biocal 370 (Medtronic, Minneapolis, MN), Lifebridge (Zoll Lifebridge, Germany), and Sorin Lifebox (Sorin Group, Munich, Germany). The systems comprised a centrifugal pump, membrane oxygenator, heat exchanger, and bypass cannulas. 

### 2.5. ECPR Management

To ensure continuous quality of ECPR therapy, a team, consisting of cardiologists, cardiac surgeons, and cardiopulmonary technicians, as well as allied professionals was formed. They were trained to set up and manage the ECPR systems to enable 24/7 service. On admission, patients were transferred directly to the catheterization laboratory for ECPR implantation. During night or weekend shifts the patients were firstly brought under ongoing resuscitation to the emergency department or intensive care unit and then transferred to the catheterization laboratory. The femoral vein and artery were percutaneously cannulated using a modified Seldinger technique. To prevent a lower limb ischemia, a perfusion canula was placed distally into the entry site of the arterial cannula. Unfractioned heparin was administered (100 IU/kg) to maintain an activated clotting time of 160–200 s. After hemodynamic and respiratory stabilization of the patient, coronary angiography and, if required, percutaneous myocardial revascularization was performed. Subsequently, the patients were admitted to the intensive care unit, and in all patients, therapeutic hypothermia was induced (target body temperature was 33 °C) during the first 24 h.

### 2.6. Statistical Analysis

Statistical analysis was performed using SPSS statistics version 23 (IBM, Armonk, NY, USA) and MedCalc Statistical Software version 18.11.3 (MedCalc Software bvba, Ostend, Belgium; https://www.medcalc.org; 2019). The primary endpoint was 30-day mortality. The continuous variables had mostly non-normal distributions and are presented for reasons of uniformity as medians with interquartile range (IQR). Therefore, a Mann–Whitney U test was used. The categorical variables are reported as numbers and percentages and were compared by Chi-squared test. The impact on 30-day mortality was assessed by uni- and multivariable Cox regression analyses. Variables with univariate association to 30-day mortality with a *p*-value of <0.1 at every individual point of time were included in the subsequent multivariable analysis. Receiver-operating curves were analyzed and area under the curve (ROC-AUC) calculated, and optimal cut-off values were calculated by means of the Youden Index. The survival rate was investigated by Kaplan–Meier curves, and a log-rank-test was used to compare the groups. All the tests were 2-tailed and a *p*-value of <0.05 was considered to be statistically significant. Furthermore, a binary logistic regression was performed to investigate whether baseline parameters and lactate clearance predict the neurological outcome assessed by GOS. The scores of the GOS were pooled into two groups: scores of 1–3 (dead or poor outcome) and scores of 4–5 (good outcome). Therefore, variables with univariate association for the neurological outcome of with a *p*-value of <0.1 were included.

## 3. Results

### 3.1. Baseline Characteristics

Baseline characteristics of the study population are presented in Table 1. A total of 93 patients were included in the study. The mean age was 57 years (interquartile range (IQR) of 19) and there was a predominance of male patients (76%). The overall survival rate after 30 days was 28% (26 of 93). 14% (9 of 65) of the patients had a good neurological outcome with a GOS score of ≥4. The median duration of resuscitation was 35 (IQR 45) min.

Comparing survivors and non-survivors, there was no major difference in clinical profile (age, sex, and body mass index (BMI)) at the time of admission to the intensive care unit (ICU). Survivors were not dissimilar with regards to comorbidities, such as peripheral arterial disease, coronary artery disease, prior myocardial infarction, diabetes mellitus, and coronary artery bypass graft (CABG), as well as chronic obstructive pulmonary disease (COPD) and prior strokes, compared with non-survivors, as presented in Table 1. Catecholamines were used less frequently in survivors during the first 24 h (noradrenalin: 58% of survivors vs. 90% of non-survivors; *p* = 0.001; dobutamine: 31% of survivors vs. 66% of non-survivors, *p* = 0.005), as presented in Table 1. The ECPR flow did not differ between survivors and non-survivors (Table 1). Survivors and non-survivors showed differences in absolute serum lactate 6, 12, and 24 h after ECPR commencement, as presented in Table 1. Of note, we did not detect a distinction regarding lactate clearance. 

In an analysis of the bad-GOS group versus good-GOS group, there was not any difference concerning age, sex, or BMI (Table 2). The bad-GOS group trended to show longer CPR duration (40 min vs. 30 min; *p* = 0.07) and higher lactate levels at admission (11 mmol/L vs. 5 mmol/L; *p* = 0.01; Table 2).

### 3.2. Prognostic Value of Baseline Parameters and Lactate Clearance for 30-Day Mortality

**Figure 1:**
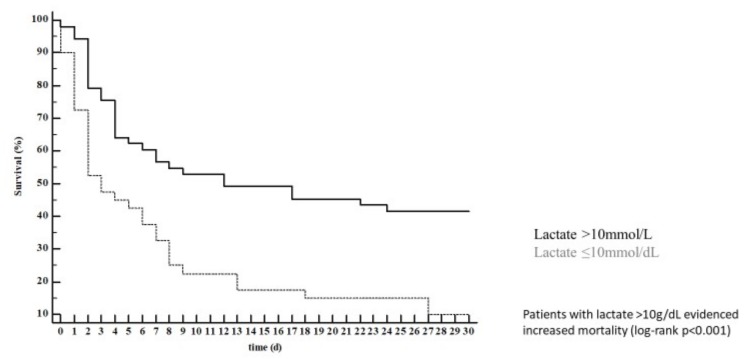
Patients with lactate >10 g/dL showed increased mortality (log-rank *p* < 0.001).

**Figure 2:**
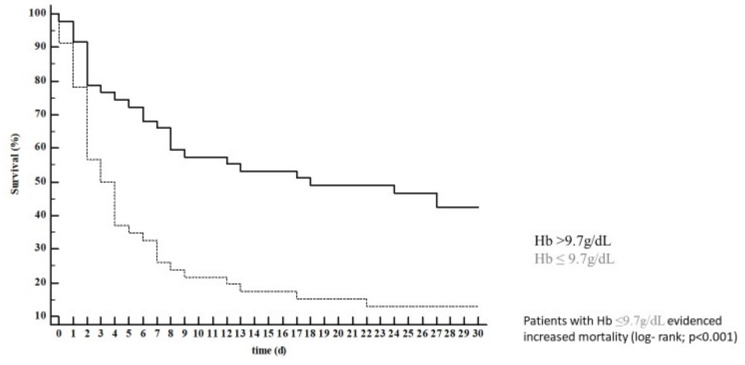
Patients with hemoglobin (Hb) ≤9.7 g/dL showed increased mortality (log- rank; *p* < 0.001).

In univariable Cox regression analysis, only serum lactate concentration (HR 1.09; 95% CI 1.04–1.13; *p* < 0.001), hemoglobin (HR 0.87; 95% CI 0.79–0.96; *p* = 0.004; this means that an increase of 1g/dL in hemoglobin is associated with a ~10% risk reduction), pH (HR 0.29 95% CI 0.15–0.55; *p* < 0.001; this means that an increase of 1 unit of pH is associated with a ~70% risk reduction), and catecholamine use were associated with 30-day mortality. All these parameters, except for pH, remained predictive of 30-day mortality after correction in a multivariable model (Table 3). The optimal cut-offs for lactate (lactate > 10 mmol/L; Figure 1) and hemoglobin (Hb ≤ 9.7 g/dL; Figure 2) were calculated. Of note, hemoglobin concentration remained associated with 30-day mortality (HR 0.87; 95% CI 0.79–0.95; *p* = 0.04) even after manually forcing the numbers of erythrocyte concentrates received during the ICU stay in the multivariable model. Lactate clearance was not associated with mortality at any timepoint (i.e., after 6, 12, and 24 h). Of note, OHCA/IHCA was not associated with 30-day mortality and neither were bystander basic life support (BLS), ventricular fibrillation, or acute myocardial infarction as the underlying pathology (Table 3).

### 3.3. Prognostic Value of Baseline Parameters and Lactate Clearance for Bad GOS Scores

**Figure 3:**
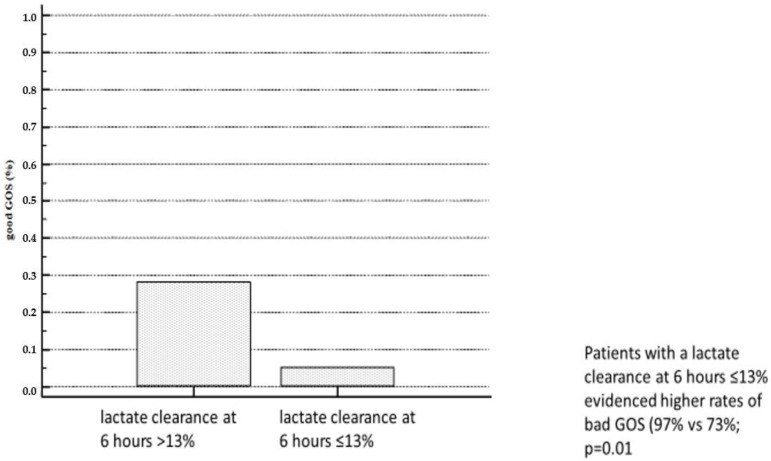
Patients with a lactate clearance at 6 h ≤13% showed higher rates of bad GOS (97% vs. 73%; *p* = 0.01.

In univariable logistic regression, only lactate concentration (OR 1.20; 95% CI 1.01–1.44; *p* = 0.04) and lactate clearance after 6 h (OR 0.97; 95% CI 0.94–0.99; *p* = 0.02) and 12 h (OR 0.98; 95% CI 0.95–0.99; *p* = 0.04) were associated with good GOS scores (Table 4). In a multivariable model of all the evaluated variables, only lactate clearance (after 6 h; OR 0.97; 95% CI 0.94–0.997; *p* = 0.03) was associated with good GOS scores. Evaluating only patients with an initial lactate above 2.0 mmol/L did not change this association of lactate clearance and GOS score (0.968; 95% CI 0.942–0.995; *p* = 0.02). The optimal cut-off of lactate clearance at 6 h for the prediction of bad GOS scores was at ≤13%. Patients with a lactate clearance at 6 h ≤13% evidenced higher rates of bad GOS scores (97% vs. 73%; *p* = 0.01; Figure 3) but 30-day mortality was statistically not dissimilar (80% vs. 63%; *p* = 0.10).

## 4. Discussion

In this multicenter study of CA treated with ECPR, the survival rate after 30 days was 27%, with only 14% of the cohort presenting a good neurological outcome. Absolute serum lactate and hemoglobin qualified as a valuable marker to predict 30-day mortality in these patients. Interestingly, although lactate clearance was not associated with 30-day mortality, it was predictive of neurological outcome as assessed by the GOS.

The current study is consistent with previous studies with respect to mortality rates: Dennis and coworkers recently presented a study of patients treated with ECPR following in- and out-of-hospital CA [8]. Haneya et al. also presented data from a study treating both patients with in- and out-of-hospital CA with ECPR [16]: 34% of their patients survived to hospital discharge (93% without severe neurologic deficit). Although Haneya et al. found a worse outcome in patients treated with ECPR following OHCA compared to in-hospital CA (IHCA), this was not the case in the study by Dennis et al. [16,17,18]. In our study, we also found no difference in mortality rates between IHCA and OHCA. Furthermore, the median CPR duration did not differ between survivors and non-survivors. Patients with bad GOS scores tended to show longer median CPR duration. 

Because mortality rates remain high and ECPR treatment is resource intensive, adequate assessment of prognosis estimation is of utmost importance for both initiation or early termination of ECPR treatment. Several potentially important parameters have been studied: There was no difference in blood product use, ECLS flow rates, and ECLS running time between survivors and non-survivors. None of the comorbidities differed between survivors and non-survivors. 

Anemia constitutes the most frequent concomitant pathology in critical care patients, affecting 60–80 percent of patients admitted to the ICU [14,19,20]. In our study, in the cohort of patients undergoing ECPR, hemoglobin was inversely associated with mortality. This association remained even after correction for co-founders in a multivariable model. Even after the manual forcing of the numbers of erythrocyte concentrates (ECs) obtained during the ICU stay into the model, the association of anemia and mortality persisted. In intermittent hypoxia, as during cardiac arrest, anemia represents an important co-factor fostering hypoxic development through the reduced provision of oxygen, which might contribute to this finding [19,21].

In clinical routine, serum lactate levels are established to evaluate macro- and microvascular perfusion and oxygenation [22,23]. Whether serum lactate qualifies as a predictive value in the ECPR population has been a subject of debate. Of note, Leick et al. found no association between elevated lactate and increased mortality in patients with OHCA undergoing in-hospital ECLS treatment [3]. In patients suffering from myocarditis treated with ECLS, lactate concentrations after 24 h, in addition to peak troponin levels, were associated with mortality [24]. Nevertheless, a retrospective study found similar results to our findings of predictive lactate levels for mortality in ECPR-treated patients [8,9]. In a study evaluating ST-segment elevation myocardial infarction (STEMI) patients suffering from severe cardiogenic shock treated with ECLS, lactate clearance at 24 h was associated with mortality, in addition to another biomarker (blood urea nitrogen), as were BMI and prolonged door-to-balloon times [25]. Thus, substantial evidence has shown that lactate is a strong prognostic marker for 30-day survival [1], and here, the calculated baseline lactate threshold was 10 mmol/l, therefore confirming these earlier findings [26]. 

Mehta et al. revealed that the neurological outcome in patients treated with ECPR is dependent on various risk factors, such as hypoxia and hypotension, as well as acidosis and cardiac output [26,27]. In our study, we found an association between low lactate levels and a good neurological outcome, which confirms the previous results of Müllner et al. [28]. Interestingly, in a multivariable model, lactate clearance, but not lactate concentration remained predictive of good GOS scores. This might indicate that prolonged tissue hypoperfusion contributes to neurological damages. Lactate clearance is hypothesized to provide further insight in the effectiveness and outcome of patients treated with ECLS. Substantial evidence shows that lactate clearance better predicts the outcomes of critically ill patients as compared with the lactate level [29]. Despite these findings, our multivariable analysis attributed lactate clearance with no value with respect to its ability to predict 30-day mortality over lactate level. Nevertheless, our results might have value in the decision process regarding when to stop ECPR considering not only mortality but also neurological outcome. 

### Limitations

Our study has several limitations. First, it does not represent a prospective study with clear inclusion and exclusion criteria, and the percentage of ECPR use was not reported. Patients that undergo ECPR might be influenced by a selection bias, making it more likely that younger patients in worse circumstances receive ECPR. Second, not all the data of all the patients were complete, because scenarios differ and the focus has been placed on patient treatment. Thus, the data were incomplete, no echocardiographic data were available, and no information about further treatment (e.g., about revascularization) was available. Third, because the numbers of ECPR cases are infrequent, the timespan of patient treatment is rather long, and the absolute patient numbers are low, which might also contribute to the surprising finding that lactate clearance after 6 h, but not after 12 and 24 h, was associated with neurologic outcome. However, we believe in presenting this important and clinically relevant real-world data.

## 5. Conclusions

Absolute serum lactate and hemoglobin at the baseline qualify as valuable markers to predict mortality in patients with ECPR. Although lactate clearance does not predict mortality, it was the sole predictor of good neurological outcomes and might, therefore, guide clinicians when to stop ECPR.

## Figures and Tables

**Table 1:** Baseline characteristics of the study population.

	Overall		Total	Survivor		Total	Non-Survivor		Total	
	Median	IQR	*n* =	Median	IQR	*n* =	Median	IQR	*n* =	*p* =
Age (years)	57.00	19.00	93	51.50	25.50	26	57.00	16.00	67	0.17
BMI (kg/m^2^)	26.37	4.58	64	26.23	4.11	13	27.34	5.27	51	0.17
CPR Duration (min)	35.00	45.00	67	30.00	37.50	20	40.00	50.00	47	0.52
Baseline C-reactive protein (mg/L)	13.00	40.10	63	9.40	44.35	13	13.60	41.48	50	0.49
Baseline serum creatine (µmol/L)	9.82	3.06	93	10.70	3.09	26	9.34	3.06	67	0.02
Baseline hemoglobin (g/dL)	1.33	1.05	93	1.24	0.54	26	1.50	1.23	67	0.17
Baseline serum lactate (mmol/L)	9.30	8.30	93	6.30	6.17	26	11.40	7.80	67	<0.001
Lactate 6 h (mmol/L)	8.00	9.75	93	5.45	6.81	26	9.80	9.80	67	0.001
Lactate 12 h (mmol/L)	6.07	8.60	86	3.75	4.78	26	7.55	10.65	60	0.01
Lactate 24 h (mmol/L)	4.33	5.08	80	3.70	3.30	25	4.70	6.00	55	0.01
Lactate Clearance 6 h (%)	11.76	53.19	93	21.97	50.22	26	6.38	56.03	67	0.42
Lactate Clearance 12 h (%)	22.58	72.08	86	36.20	78.94	26	21.08	66.08	60	0.49
Lactate Clearance 24 h (%)	46.33	54.13	80	46.15	54.94	25	46.51	55.75	55	0.98
Baseline ALAT (µmol/ls)	2.87	4.04	60	1.47	4.13	13	3.02	3.75	47	0.22
Baseline Troponin (ng/L)	1212.00	8396.00	79	1580.00	8285.00	20	723.00	10,688.75	59	0.37
ECs total (n)	12.00	16.00	92	15.00	24.75	26	12.00	15.25	66	0.36
FFP total (n)	4.00	16.00	93	6.50	18.50	26	2.00	12.00	67	0.20
ECMO flow 12 h (L/min)	4.50	1.44	74	4.54	1.83	18	4.50	1.37	56	0.87
	*n* =	%	*n* =	*n* =	%	*n* =	*n* =	%	*n* =	*p* =
IHCA	39	60%	65	8	62%	13	31	60%	52	1.00
Female	22	24%	93	5	19%	26	17	25%	67	0.60
Peripheral arterial disease	8	9%	91	1	4%	24	7	10%	67	0.68
Coronary artery disease	68	76%	90	19	76%	25	49	75%	65	1.00
Prior myocardial infarction	8	13%	64	1	8%	13	7	14%	51	1.00
Prior CABG	3	5%	65	0	0%	13	3	6%	52	1.00
Diabetes mellitus	21	24%	89	4	16%	25	17	27%	64	0.41
Chronic renal failure	12	18%	65	3	23%	13	9	17%	52	0.69
Prior stroke	6	9%	65	2	15%	13	4	8%	52	0.59
Noradrenalin 1 d	75	81%	93	15	58%	26	60	90%	67	0.001
Dobutamin 1 d	52	56%	93	8	31%	26	44	66%	67	0.005
Suprarenin 1 d	43	46%	93	15	58%	26	28	42%	67	0.25

BMI: body mass index; CPR: cardiopulmonary resuscitation; ALAT: alanine aminotransferase; ECs: erythrocyte concentrates; FFP, fresh frozen plasma; IHAC: in-hospital cardiac arrest; CABG: coronary artery bypass graft; ECMO, extracorporeal membrane oxygenation; IQR, interquartile range.

**Table 2:** Baseline characteristics of the study population.

	Good GOS		Total	Bad GOS		Total	
	Median	IQR	*n* =	Median	IQR	*n* =	*p* =
Age (years)	60.00	25.50	9	57.00	16.00	56	0.90
BMI (kg/m^2^)	25.95	6.05	9	26.81	5.24	55	0.27
CPR Duration (min)	30.00	25.00	7	40.00	48.75	34	0.07
Baseline C-reactive protein (mg/L)	9.40	42.10	9	13.60	41.48	54	0.31
Baseline serum creatine (µmol/L)	10.79	4.11	9	9.10	3.34	56	0.11
Baseline hemoglobin (g/dL)	1.12	0.60	9	1.35	1.07	56	0.08
Baseline serum lactate (mmol/L)	4.60	5.40	9	11.25	8.65	56	0.01
Lactate 6 h (mmol/L)	3.20	4.00	9	8.40	10.15	56	0.003
Lactate 12 h (mmol/L)	2.20	3.65	9	5.93	11.75	49	0.01
Lactate 24 h (mmol/L)	1.90	3.38	8	4.55	8.15	44	0.01
Lactate Clearance 6 h (%)	33.33	46.20	9	5.18	58.78	56	0.01
Lactate Clearance 12 h (%)	59.15	45.75	9	22.94	59.65	49	0.01
Lactate Clearance 24 h (%)	63.37	51.59	8	40.29	63.98	44	0.09
Baseline ALAT (µmol/ls)	1.47	3.00	9	3.02	3.94	51	0.21
Baseline Troponin (ng/L)	729.00	4044.00	7	1510.00	12.40	47	0.31
RCB total (n)	6.00	13.50	9	11.50	12.75	56	0.28
FFP total (n)	0.00	1.00	9	0.00	7.50	56	0.11
ECMO flow 12 h (L/min)	4.55	2.74	6	4.50	1.00	44	0.72
	*n* =	%	*n* =	*n* =	%	*n* =	*p* =
IHCA	6	67%	9	33	59%	56	0.73
Female	2	22%	9	14	25%	56	1.00
Peripheral arterial disease	0	0%	9	4	7%	56	1.00
Coronary artery disease	8	89%	9	39	70%	56	0.43
Prior myocardial infarction	0	0%	9	8	15%	55	0.59
Prior CABG	0	0%	9	3	5%	56	1.00
Diabetes mellitus	1	11%	9	15	27%	56	0.43
Chronic renal failure	2	22%	9	10	18%	56	0.67
Prior stroke	1	11%	9	5	9%	56	1.00
Noradrenalin 1d	9	78%	9	50	89%	56	0.31
Dobutamin 1d	6	67%	9	44	79%	56	0.42
Suprarenin 1d	2	22%	9	18	32%	56	0.71

GOS: Glasgow Outcome Scale; ECs: erythrocyte concentrates.

**Table 3:** Univariable and multivariable analysis of parameters predicting 30-day mortality.

	Univariable			Multivariable		
	HR	95% CI	*p*-Value	HR	95% CI	*p*-Value
Serum lactate (baseline)	1.09	1.04–1.13	<0.001	1.10	1.05–1.15	<0.001
Serum creatine (baseline)	1.001	0.99–1.002	0.3			
C-reactive protein (baseline)	0.998	0.966–1.031	0.92			
ASAT (baseline)	1.007	0.996–1.018	0.23			
Troponin (baseline)	1.00	0.999–1.002	0.59			
Hemoglobin (baseline)	0.87	0.79–0.96	0.004	0.86	0.78–0.95	0.002
Lactate Clearance (after 6 h)	0.998	0.992–1.003	0.44			
Lactate Clearance (after 12 h)	0.996	0.991–1.002	0.19			
Lactate Clearance (after 24 h)	0.998	0.992–1.004	0.47			
Noradrenalin use	3.05	1.38–6.70	0.006	2.40	1.06–5.44	0.04
Dobutamin use	2.15	1.29–3.57	0.003	1.78	1.05–3.04	0.03
Suprarenin use	0.68	0.42–1.10	0.11			
Bystander BLS	0.86	0.37–2.02	0.73			
OHCA	1.35	0.78–2.35	0.28			
AMI	0.96	0.56–1.67	0.9			
Age	1.01	0.995–1.031	0.16			
Male sex	1.07	0.62–1.86	0.81			
CPR duration	1.003	0.997–1.010	0.26			
Ventricular fibrillation	0.78	0.45–1.35	0.36			
pH (baseline)	0.29	0.15–0.55	<0.001	0.42	0.16–1.10	0.08

OHCA: out-of-hospital cardiac arrest; CPR: cardiopulmonary resuscitation; AMI: acute myocardial infarction; BLS: basic life support. HR, hazard ratio; CI, confidence interval.

**Table 4:** Univariable and multivariable analysis of parameters predicting the neurological outcome based on Glasgow Outcome Scale.

	Univariable			Multivariable		
	OR	95% CI	*p*-Value	OR	95% CI	*p*-Value
Serum lactate (baseline)	1.2	1.01–1.44	0.04	1.26	0.98–1.62	0.08
Serum creatine (baseline)	1.004	0.995–1.012	0.4			
C-reactive protein (baseline)	1.05	0.90–1.23	0.52			
ASAT (baseline)	1.26	0.98–1.62	0.07	1.22	0.96–1.54	0.11
Troponin (baseline)	1.01	0.992–1.018	0.44			
Hemoglobin (baseline)	0.79	0.60–1.05	0.11			
Lactate Clearance (after 6 h)	0.97	0.94–0.99	0.02	0.97	0.94–0.997	0.02
Lactate Clearance (after 12 h)	0.98	0.95–0.999	0.04	not included		
Lactate Clearance (after 24 h)	0.98	0.95–1.006	0.12			
Noradrenalin use	2.38	0.40–14.19	0.34			
Dobutamin use	1.83	0.40–8.43	0.44			
Suprarenin use	1.66	0.31–8.79	0.55			
Bystander BLS	1.14	0.12–10.57	0.91			
OHCA	1.5	0.34–6.61	0.59			
AMI	0.67	0.15–2.94	0.59			
Age	1	0.96–1.06	0.87			
Male sex	1.17	0.22–6.28	0.86			
CPR duration	1.04	0.99–1.08	0.11			
Ventricular fibrillation	0.60	2.48	0.48			

## List of Abbreviations

ACS	Acute coronary syndrome
ASAT	Aspartate aminotransferase
BMI	Body mass index
CA	Cardiac arrest
CABG	Coronary artery bypass graft
COPD	Chronic obstructive pulmonary disease
CPC	Cerebral performance category
CPR	Cardiopulmonary resuscitation
ECPR	Extracorporeal cardiopulmonary resuscitation
ECMO	Extracorporeal membrane oxygenation
HR	Hazard ratio
IHCA	In-hospital cardiac arrest
OD	Odds Ratio
OHCA	Out-of-hospital cardiac arrest
PCI	Percutaneous coronary intervention
ROSC	Return of spontaneous circulation conventional
AMI	Acute myocardial infarction
BLS	Basic life support
